# Combination CD200R/PD-1 blockade in a humanised mouse model

**DOI:** 10.1093/immadv/ltad006

**Published:** 2023-03-30

**Authors:** Martin Fellermeyer, Consuelo Anzilotti, Christopher Paluch, Richard J Cornall, Simon J Davis, Uzi Gileadi

**Affiliations:** MRC Human Immunology Unit, John Radcliffe Hospital, University of Oxford, Oxford, UK; Radcliffe Department of Medicine, John Radcliffe Hospital, University of Oxford, Oxford, UK; MRC Human Immunology Unit, John Radcliffe Hospital, University of Oxford, Oxford, UK; Radcliffe Department of Medicine, John Radcliffe Hospital, University of Oxford, Oxford, UK; Nuffield Department of Medicine, Henry Wellcome Building for Molecular Physiology, University of Oxford, Oxford, UK; MRC Human Immunology Unit, John Radcliffe Hospital, University of Oxford, Oxford, UK; Radcliffe Department of Medicine, John Radcliffe Hospital, University of Oxford, Oxford, UK; Nuffield Department of Medicine, Henry Wellcome Building for Molecular Physiology, University of Oxford, Oxford, UK; MRC Human Immunology Unit, John Radcliffe Hospital, University of Oxford, Oxford, UK; Nuffield Department of Medicine, Henry Wellcome Building for Molecular Physiology, University of Oxford, Oxford, UK; CAMS Oxford Institute, Henry Wellcome Building for Molecular Physiology, University of Oxford, Oxford, UK; MRC Human Immunology Unit, John Radcliffe Hospital, University of Oxford, Oxford, UK; Radcliffe Department of Medicine, John Radcliffe Hospital, University of Oxford, Oxford, UK; MRC Human Immunology Unit, John Radcliffe Hospital, University of Oxford, Oxford, UK; Radcliffe Department of Medicine, John Radcliffe Hospital, University of Oxford, Oxford, UK

**Keywords:** PD-1, CD200R, nivolumab, cancer immunotherapy, monoclonal antibody

## Abstract

There is an increasing number of immune-checkpoint inhibitors being developed and approved for cancer immunotherapy. Most of the new therapies aim to reactivate tumour-infiltrating T cells, which are responsible for tumour killing. However, in many tumours, the most abundant infiltrating immune cells are macrophages and myeloid cells, which can be tumour-promoting as well as tumouricidal. CD200R was initially identified as a myeloid-restricted, inhibitory immune receptor, but was subsequently also found to be expressed within the lymphoid lineage. Using a mouse model humanised for CD200R and PD-1, we investigated the potential of a combination therapy comprising nivolumab, a clinically approved PD-1 blocking antibody, and OX108, a CD200R antagonist. We produced nivolumab as a murine IgG1 antibody and validated its binding activity *in vitro* as well as *ex vivo*. We then tested the combination therapy in the immunogenic colorectal cancer model MC38 as well as the PD-1 blockade-resistant lung cancer model LLC1, which is characterised by a large number of infiltrating myeloid cells, making it an attractive target for CD200R blockade. No significant improvement of overall survival was found in either model, compared to nivolumab mIgG1 monotherapy. There was a trend for more complete responses in the MC38 model, but investigation of the infiltrating immune cells failed to account for this. Importantly, MC38 cells expressed low levels of CD200, whereas LLC1 cells were CD200-negative. Further investigation of CD200R-blocking antibodies in tumours expressing high levels of CD200 could be warranted.

## Graphical Abstract

**Graphical Abstract:**
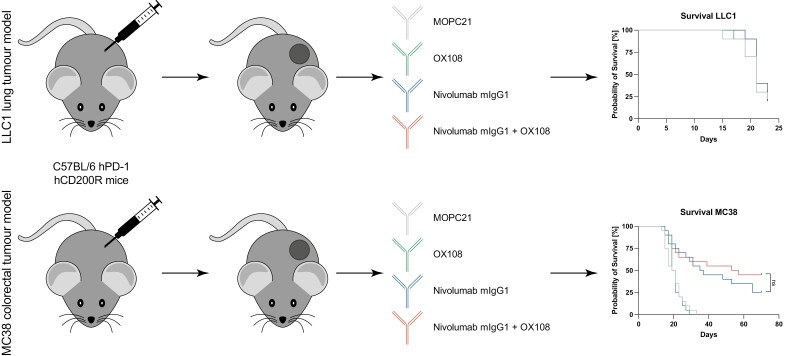

## Introduction

Monoclonal antibodies blocking inhibitory immune receptors have revolutionised cancer treatment in recent years. While most of those therapies aim to reactivate T cells to induce tumour killing, there has been growing interest in targeting other cell types present in the tumour microenvironment (TME), including myeloid cells [1]. A great number of approaches are being utilised, including the use of immune-checkpoint inhibitors to trigger phagocytosis [2], or to induce the re-programming of myeloid-derived suppressor cells (MDSCs) and tumour-associated macrophages [3, 4].

CD200R is an inhibitory immune receptor predominantly found on myeloid cells [5]. In mice, loss of its ligand, CD200, results in enhanced disease in autoimmune models including experimental autoimmune encephalomyelitis and collagen-induced arthritis [5]. While there is a substantial body of evidence that the CD200/CD200R axis is important for myeloid immunoregulation in mice [6–9] and humans [10–12], the role in cancer is more complex. Some studies showed that activation of CD200R, either by binding of the ligand CD200 or through treatment with an agonistic antibody, leads to inhibition of tumour-associated myeloid cells (TAMCs), ultimately reducing tumour burden [13, 14]. However, other studies indicated that CD200/CD200R engagement can lead to the expansion of MDSCs, which is tumour-promoting [15, 16]. The inflammatory milieu within the TME is likely to be one of the determining factors controlling the overall effect of CD200R signalling [17, 18]. It is also important to note that while most inhibitory effects of CD200R have been studied in myeloid cells, lymphoid lineage cells also express CD200R [19], and inhibitory effects in this setting have been well documented [20–22]. In an *in vitro* model, CD200R signalling resulted in the upregulation of PD-1 on CD8^+^ T cells, establishing the first link between the two inhibitory receptors [23]. Triggering of both receptors simultaneously, through their respective ligands, led to the synergistic inhibition of T-cell activation [23].

PD-1 checkpoint therapy is remarkably successful in a variety of tumour types and there is also considerable interest in combining it with orthogonal reagents. In this study, we tested the combination of antagonistic PD-1 and CD200R antibodies, using a ‘murinised’ version of nivolumab (mNivo) as well as clone OX108, a well-established blocking antibody of the CD200/CD200R interaction [24]. Using double knock-in mice that express humanised forms of the extracellular regions of PD-1 and CD200R, allowed us to test anti-human antibodies reactive with these receptors *in vivo*. We established two different mouse tumour models, MC38 and LLC1, which are sensitive and resistant to anti-PD-1 monotherapy, respectively. Blocking PD-1 and CD200R did not result in any significant survival improvement over mNivo monotherapy. However, a trend towards more complete responses in the MC38 model warrants further investigation. Unlike LLC1 cells, MC38 cells expressed low levels of CD200, which suggests a potential explanation for the observed effect.

## Materials and methods

### Mice and cell lines

C57BL/6JOlaHsd mice were humanised for CD200R and PD-1 as described in Supplementary Fig. S1A and B. Each strain was designed to express the human extracellular domain as a chimaera with the murine transmembrane and cytoplasmic domains. The production was outsourced to InGenious Targeting Laboratory and Taconic Biosciences for the human knock-in CD200R and PD-1 mouse, respectively. Humanised CD200R and humanised PD-1 mice were crossbred until homozygosity for both transgenes. Double knock-in humanised CD200R and PD-1 mice were bred and maintained at the Biomedical Services (University of Oxford) in accordance with Home Office guidelines. Depending on the original sex of the cancer cell line, female or male mice between 6 and 14 weeks of age were used for MC38 and LLC1 tumour models, respectively.

Cell lines were grown at 37°C in a 5% CO_2_ atmosphere in a humidified incubator. MC38 cells (kindly provided by Stephen Beers, University of Southampton) were maintained in RPMI 1640 supplemented with 10% fetal calf serum (FCS), 2 mM L-glutamine, and 1 mM sodium pyruvate. Lewis lung carcinoma (LLC1) cells (kindly provided by Vincenzo Cerundolo, University of Oxford) were maintained in RPMI 1640 supplemented with 10% FCS and 2 mM L-glutamine. No antibiotics were used for cells that were used for animal experiments and cells were validated by STR profiling (IDEXX BioAnalytics).

Human Jurkat T cells and mouse BW5147 T cells (both kindly provided by Peter Steinberger, University of Vienna) were cultured in RPMI 1640 supplemented with 10% FCS, 2 mM L-glutamine, 100 U/ml penicillin, and 100 µg/ml streptomycin. HEK293T cells were maintained in DMEM supplemented with 10% FCS, 2 mM L-glutamine, 10 mM HEPES, 100 U/ml penicillin, and 100 µg/ml streptomycin. CHO-K1 cells were cultured in DMEM with 10% FCS, 2 mM L-glutamine, 1 mM sodium pyruvate, 100 U/ml penicillin, and 100 µg/ml streptomycin.

### Antibody production

All antibodies (MOPC21 isotype control, nivolumab, OX108) were designed as mIgG1 isotype with a D265A mutation to reduce Fc gamma receptor (FcγR) binding [25]. The heavy chain constant region included mutations that improve Protein A binding [26] without affecting FcRn binding (personal communication by Absolute Antibodies Ltd.). Heavy and light chains were ligated into pHR-SIN-IRESEm. HEK293T cells were co-transfected with the respective pHR plasmid as well as pmDG and p89.1 plasmids for lentivirus production. Subsequently, CHO-K1 cells were transduced with equal amounts of viruses containing the respective heavy and light chains. Once cells expanded, the medium was switched to 1% ultra-low IgG FCS (ThermoFisher) with additional supplementation of 1 × non-essential amino acids (ThermoFisher). Supernatants were harvested every week and affinity-purified using Protein A columns (Cytiva). All antibodies were size-excluded using a ProteoSEC 16/60 6-600 HR SEC column on an endotoxin-free ÄKTA start machine. All antibodies were confirmed to be endotoxin-free using the Pierce Chromogenic Endotoxin Quant Kit (ThermoFisher).

### Surface plasmon resonance (SPR)

Antibody affinities were measured using a Biacore 8K (Cytiva). A Protein A chip was used to capture the respective antibodies (69.76 nM) in the corresponding flow cells. hPD-1-2xHis and hCD200R-mFc-His were affinity purified using Ni-NTA columns and size-excluded to remove aggregates. Analytes were injected in 1:3 dilutions as specified in the figures. For the single-cycle experiment concentrations ranging from 2777.78 to 1.27 nM were used. All runs were reference and blank subtracted. All proteins were diluted in HBS-EP+ running buffer (Cytiva) and all measurements were undertaken at 37°C. For analysis of CD200R-mFc-His binding to OX108, the Protein A chip was blocked by the injection of high concentrations of MOPC21 (697.58 nM for 300 s) into both flow cells to occupy all Protein A binding sites. This approach was validated by running mNivo as a control in another channel, which did not demonstrate significant binding upon hCD200R-mFc-His injection. Affinity constants were derived by fitting the curves with a 1:1 binding model (Biacore Insight Evaluation Software, Cytiva).

### *In vitro* T-cell activation assay

Jurkat reporter cells expressing GFP under an NFκB promoter (kindly provided by Peter Steinberger, University of Vienna) [27] were transduced with PD-1 or a chimeric protein comprising the CD200R extracellular domain and PD-1 cytosolic signalling domain. BW5147 cells (T-cell stimulator (TCS) cells, kindly provided by Peter Steinberger, University of Vienna) expressing a membrane-bound anti-CD3 (OKT3) single-chain variable fragment (scFv) were transduced with PD-L1 or CD200. For the T-cell activation assay, 1.5 × 10^5^ Jurkat T cells expressing PD-1 or CD200R were co-cultured with 1.5 × 10^5^ TCS cells expressing the respective ligand in a 96-well U-bottom plate. Blocking antibodies or MOPC21 isotype control were added at varying concentrations and incubated in a humidified incubator at 37°C and 5% CO_2_. After 24 h, cells were spun down and stained with anti-mCD45-PE to gate out the TCS. GFP was measured by flow cytometry, indicating NFκB transcriptional activation. Fold changes were calculated by dividing all values from the co-cultures by the background of the Jurkat-only culture.

### Stimulation of splenocytes

Spleens from double knock-in mice were harvested and processed in RPMI 1640 with 10% FCS. Spleens were mashed through a 70 µm cell strainer, resuspended in red blood cell lysis buffer (Qiagen) and counted. A total of 2 × 10^6^ splenocytes were stimulated overnight with PMA/ionomycin (ThermoFisher) and subsequently incubated for 20 min with the respective blocking antibody. Flow cytometry was used to determine blocking efficiency.

### *In vitro* IFN-γ stimulation

A total of 2 × 10^5^ cells were seeded in a six-well plate. The next day, media containing varying concentrations of IFN-γ was added. After 24 h, cells were analysed for MHC-I, PD-L1, and CD200 expression using flow cytometry.

### Subcutaneous tumour model

C57BL/6 double knock-in mice were injected subcutaneously with syngeneic cancer cell lines. A total of 5 × 10^5^ MC38 cells and 1 × 10^5^ LLC1 cells were injected in 100 µl ice-cold PBS. Mice were anaesthetised using isoflurane, shaved and cells were injected using a 25G needle. Mice were monitored for a palpable tumour every other day and measurements (using calliper) were taken as soon as this was sufficiently accurate. Tumours were measured in all three dimensions and the volume was calculated using the following formula: $V=\frac{\pi }{6}\times length\times width\times height$

For MC38 tumours, treatment was started once the average tumour volume exceeded 40 mm^3^ (day 7/8). As LLC1 tumours initially grew slower, treatment was started once the average tumour volume exceeded 20 mm^3^ (day 9). Mice were randomised into groups (*n* = 10) to achieve a similar average tumour volume for all groups. Treatments were blinded so that the investigator measuring the tumour sizes was not aware of the different group allocations. Mice were treated four times over the course of two weeks with 200 µg/antibody/treatment. Mice were culled once tumour volume exceeded 1000 mm^3^ or an ulceration persisted over 48 h.

### Tumour and lymph node harvest and immune cell isolation

After cervical dislocation, tumours and lymph nodes (draining and non-draining) were harvested in digestion medium. Tumours were digested in HBSS containing 1 mg/ml Collagenase Type IV (ThermoFisher) and 0.1 mg/ml DNase I (Sigma). Lymph nodes were digested in HBSS containing 1 mg/ml Collagenase Type D (Sigma) and 0.1 mg/ml DNase I (Sigma). Tumours were cut into small pieces and digested for 45 min at 37°C. The cell suspension was passed over a 70 µm cell strainer and washed in PBS + 1% FCS + 2 mM EDTA. For analysis of tumour cells expressing MHC-I, PD-L1 and CD200, 2 × 10^6^ cells were used for flow cytometry staining. For isolation of tumour-infiltrating immune cells (TIICs), immune cells were enriched using Ficoll-Paque (Cytiva) density gradient centrifugation. Lymph nodes were pierced and torn using forceps, incubated for 30 min at 37°C in the digestion buffer and passed over a 70 µm cell strainer.

### Flow cytometry staining

Isolated cells were stained with antibody cocktails for subsequent flow cytometry analysis using a BD LSRFortessa X-20. A total of 2 × 10^6^ cells were used for each staining. Cells were washed with PBS and stained with Zombie NIR Live/Dead stain (ThermoFisher) for 15 min at room temperature (RT). Next, cells were incubated for 10 min on ice with a mouse Fc block (BioLegend). Subsequently, cells were resuspended in 50 µl of the respective antibody cocktail and incubated for 15 min on ice. Cells were washed with PBS and resuspended in IC Fixation Buffer (ThermoFisher) and incubated for 30 min at RT. Afterwards, cells were washed and resuspended in PBS + 1% FCS + 2 mM EDTA. FMO controls and compensation beads were included in every run and were treated in the same way as the respective samples. Quantibrite PE beads (BD Biosciences) were used according to the manufacturer’s instructions to measure levels of CD200. Samples were analysed using FlowJo 10.8.1.

Antibodies were purchased from BioLegend unless otherwise stated: hCD200R-PE (OX108, ThermoFisher), hPD-1-PE-Cy7 (EH12.2H7), CD200-PE (OX-90), CD200-APC (OX-90), PD-L1-PE (MIH5, ThermoFisher), MHC-I-eFluor450 (28-14-8, ThermoFisher), CD45.2-BV510 (104), CD11b-BV605 (M1/70), CD11c-PerCP-Cy5.5 (N418), Ly-6G-FITC (1A8), Ly-6C-BV785 (HK1.4), F4/80-eFluor450 (BM8, ThermoFisher), CD206-APC (C068C2), MHC-II-BV711 (I-A/I-E, M5/114.15.2), CD103-PE-Dazzle594 (2E7), CCR7-BUV395 (4B12, BD), hPD-1-BUV737 (EH12.1, BD), CD3-BV650 (17A2), CD4-PerCP-Cy5.5 (RM4-4), B220-B785 (RA3-6B2), NK1.1-APC (PK136), CD8-BV421 (53-6.7), CD25-BUV395 (PC61), TCR-β-PE (H57-597), CD62L-APC (MEL-14), CD44-FITC (IM7), CD8-BV711 (53-6.7), and CD69-PE-Cy7 (H1.2F3). All antibodies are anti-mouse unless otherwise stated.

The gating strategies are shown in Supplementary Figs. S5A, S6A and B for the myeloid, lymphoid, and T-cell panel, respectively.

### Statistics

Data were analysed using GraphPad Prism 9.4.1. Immune cell populations were analysed using two-way ANOVA tests with Tukey’s multiple comparisons test. Survival experiments were plotted as Kaplan–Meier curves and analysed by log-rank (Mantel–Cox) test. Complete responses were analysed by chi-square tests. Differences were considered statistically significant when *P* ≤ 0.05.

## Results

### Murinised nivolumab retains activity *in vitro* and *ex vivo*

Using double knock-in mice humanised for the extracellular regions of CD200R and PD-1 allowed us to test anti-human receptor antibodies in the mouse. The current FDA-approved blocking antibodies targeting PD-1 are all hinge-stabilised hIgG4 antibodies, which retain binding to FcγRs, but are generally considered to have limited effector functions [28]. However, there has been increasing evidence that Fc-null PD-1 blocking antibodies are more efficacious compared to their Fc-engaging counterparts [29, 30], with tislelizumab being the first anti-PD-1 antibody with reduced FcγR binding to enter phase III clinical trials [31]. To allow for the most potent therapy, therefore, we produced all our antibodies in the form of the mIgG1 isotype (i.e. with the constant region of mIgG1), which exhibits limited effector functions, combined with a D265A mutation of the CH2 region of the heavy chain, which further reduces FcγR binding [25].

**Figure 1.**
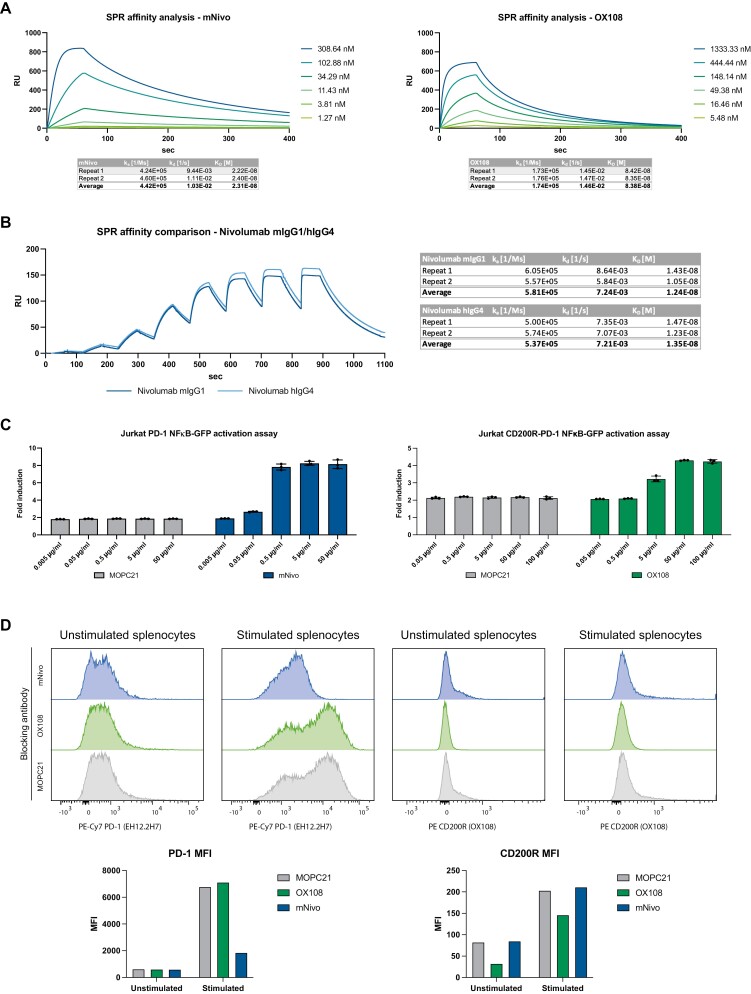
Validation of produced mIgG1 antibodies *in vitro* and *ex vivo.* (A) mNivo and OX108 were captured on a Protein A SPR chip. MOPC21 was captured in the reference channel. The corresponding protein (hPD-1-2xHis and hCD200R-mFc-His) was injected in 1:3 dilutions as specified. The Biacore Insight Evaluation Software was used to fit a 1:1 model and derived affinity constants (k_a_, k_d_, and K_D_) are shown in the table below. (B) Nivolumab mIgG1 (mNivo) and nivolumab hIgG4 were captured on a Protein A SPR chip. MOPC21 was captured in the reference channel. hPD-1-2xHis was injected in 1:3 dilutions with concentrations from 2777.78 nM to 1.27 nM. The Biacore Insight Evaluation Software was used to fit a 1:1 model and derived affinity constants (k_a_, k_d_, and K_D_) are shown in the tables. (C) Jurkat reporter cells (GFP under an NFκB promoter) expressing PD-1 or a chimeric protein CD200R-PD-1 were stimulated with TCS cells expressing PD-L1/CD200. Respective blocking antibodies or MOPC21 were added, and cells were incubated for 24 h. GFP expression was analysed by flow cytometry and normalised to Jurkat-cells-only cultures. Data are presented as mean ± standard deviation (SD). (D) Splenocytes from double knock-in mice were stimulated with PMA/ionomycin or control. After 24 h, cells were blocked using produced antibodies. Competition for binding was analysed using commercial flow cytometry antibodies. Bar charts show the MFI of the respective channels.

All our antibodies were expressed in Chinese hamster ovary cells (CHO-K1 cells). Murinised nivolumab (mIgG1, hereinafter referred to as mNivo) and OX108, the PD-1 and CD200R blocking antibodies used in this study, were demonstrated to bind to their respective target antigen using a surface plasmon resonance (SPR)-based binding assay (Fig. 1A). Importantly, a comparison of the affinities of nivolumab hIgG4 and mNivo revealed that there were no differences in the binding properties of the two antibodies (Fig. 1B). The subtle affinity differences between the multi-cycle analysis in Fig. 1A and the single-cycle analysis in Fig. 1B are likely explained by the different assay formats.

To confirm that both antibodies have the potential to activate immune cells, an *in vitro* T-cell activation assay was used wherein human Jurkat T cells express GFP under the control of an NFκB promoter [27]. These cells also expressed either full-length PD-1 or a chimeric protein consisting of the extracellular region of CD200R and the cytoplasmic domain of PD-1. The T cells were stimulated with TCS cells expressing membrane-bound anti-CD3 scFv in combination with the respective ligand, PD-L1 or CD200. The titration of both mNivo and OX108 produced dose-dependent activation of the Jurkat reporter cells (Fig. 1C).

We generated a double knock-in humanised mouse expressing human CD200R and PD-1 as a preclinical model for analysis of combination treatments using antibodies targeting these receptors. The designs of the parental mice are shown in Supplementary Fig. S1A and B. The mouse strains were validated for their expression of human PD-1 and CD200R (Supplementary Fig. S1C and E), and their naïve immune systems were shown to be unchanged versus that of C57BL/6 WT mice (Supplementary Fig. S1D and F). Importantly, murine CD200 bound to human CD200R with a similar affinity to that of mouse CD200R binding, indicating that the receptor still engages its ligand productively in humanised mice (Fig. S1G). This is known also to be the case for mouse/human PD-L1 and human PD-1 [32].

To confirm that the antibodies would bind to humanised mouse immune cells, splenocytes from the double knock-in humanised mice were isolated and stimulated for 24 h with PMA/ionomycin. After blocking with MOPC21 isotype control, OX108 or mNivo, the receptor levels of CD200R and PD-1 were measured with commercially available competing antibodies (Fig. 1D). For CD200R, fluorescently conjugated OX108 was used. For PD-1, the EH12.2H7 clone, which is known to compete with nivolumab [33], was used. As expected, only mNivo blocked the binding of EH12.2H7. Similarly, CD200R was blocked by OX108, but not MOPC21 or mNivo (Fig. 1D).

### Baseline profiling of MC38 and LLC1 cancer models

**Figure 2.**
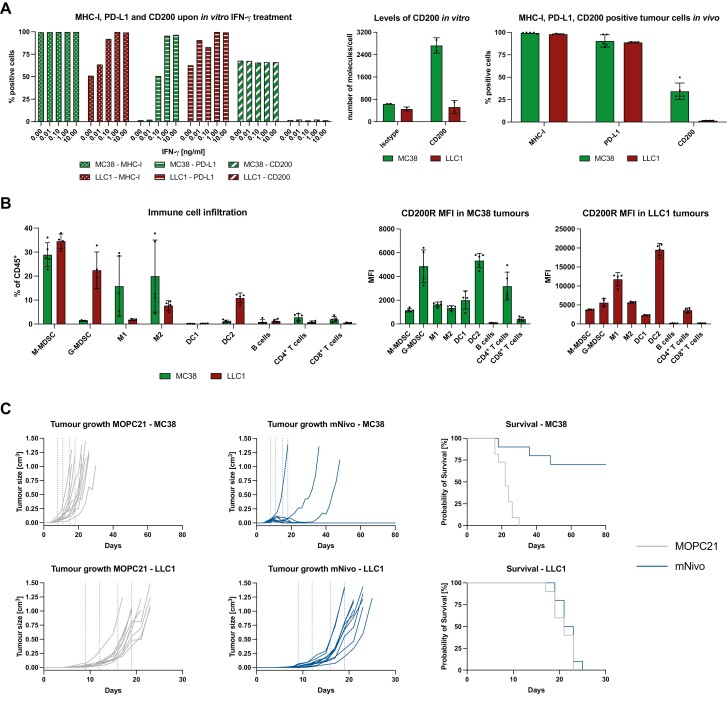
Baseline characterisation of MC38 and LLC1 cancer models. (A) *In vitro* cultured cells were stimulated with different concentrations of IFN-γ or control for 24 h (left panel). Levels of CD200 were measured using Quantibrite beads (BD Biosciences) of *in vitro* cultured cells (middle panel). After 14 days of *in vivo* growth, tumours were harvested and digested (right panel). Expression of MHC-I, PD-L1, and CD200 was analysed by flow cytometry. (B) After 14 days of *in vivo* growth, tumours were harvested and digested. Immune cells were enriched using density gradient centrifugation. The abundance of different immune cell populations was analysed (left panel) as well as the MFI of CD200R (right panel). Data are presented as mean ± SD. (C) Mice were subcutaneously injected with MC38 and LLC1 cells and tumour volume was measured every other day using calliper measurements. Once tumours reached a certain average volume (40 mm^3^ for MC38, 20 mm^3^ for LLC1), treatment with MOPC21 or mNivo was started. Mice were treated with 200 µg of antibody; four times over 2 weeks. All animal experiments were randomised and blinded. *n* = 10/group.

Next, we established the PD-1 blockade-sensitive tumour model MC38, as well as the resistant model LLC1. While MC38 is a very immunogenic model that is widely used to test new combination immunotherapies, the LLC1 tumour model stimulates a high degree of MDSC expansion [34], which made it attractive for testing CD200R blockade. First, we investigated the effects of IFN-γ on *in vitro* cultured MC38 and LLC1 cells by measuring levels of MHC-I, PD-L1, and CD200 24 h after treatment (Fig. 2A, left panel). Whereas MC38 cells exhibited dose-dependent upregulation of PD-L1, LLC1 cells expressed substantial amounts of PD-L1 prior to IFN-γ treatment. MC38 cells expressed CD200 constitutively whereas LLC1 cells did not, and expression was unaffected by IFN-γ stimulation (Fig. 2A, left panel). MC38 cells expressed approximately 2700 CD200 molecules per cell (Fig. 2A, middle panel), which is comparable to PD-L1 on immature DCs or PD-L2 on mature DCs (5724 and 5243, respectively) [35]. Both cell lines expressed high levels of MHC-I, which was further upregulated upon stimulation. In the case of MC38 cells, it was the median fluorescence intensity (MFI) that increased (data not shown).

To characterise the levels of expression of these proteins *in vivo*, as well as compare TIICs, MC38 and LLC1 cells were injected subcutaneously into homozygous double knock-in mice. After 14 days of growth, the tumours were recovered from euthanised mice, and the TIICs isolated and analysed using flow cytometry. Both tumours expressed high levels of MHC-I as well as PD-L1, whereas CD200 expression continued to be restricted to MC38 tumours (Fig. 2A, right panel). As previously reported, LLC1 tumours had large infiltrates of monocytic MDSCs (M-MDSCs) as well as granulocytic MDSCs (G-MDSCs). They also had few B and T cells as well as M1 macrophages, whereas M2 macrophages and DC2s were abundant (Fig. 2B, left panel). In contrast, in the MC38 tumour model, there were very few G-MDSCs and DC2s infiltrating the tumour, and the macrophage population was more heterogeneous with respect to the M1/M2 ratio. Although at low number, T cells were more abundant in MC38 tumours compared to LLC1 tumours (Fig. 2B, left panel). DC1s were barely detectable in either tumour model. To gain a better understanding of which immune cells could be targeted by CD200R therapy, we profiled CD200R expression levels (measured as MFI) of all identified immune cell subsets (Fig. 2B, right panel). It needs to be noted that the MC38 and LLC1 samples were acquired at different times, so the MFIs cannot be compared between the different models. CD200R was broadly expressed across all myeloid cells, with a slight increase in DC2s. In contrast, for lymphoid cells, only CD4^+^ T cells expressed detectable levels of CD200R.

### PD-1 blockade in the MC38 and LLC1 models

To validate the responsiveness of both models to mNivo monotherapy, MC38 and LLC1 were injected subcutaneously, and once a tumour was established (days 8 and 9 post-injection, respectively), the mice were treated with the MOPC21 isotype control or mNivo. While no difference in tumour growth and survival was observed for LLC1 tumours (Fig. 2C, bottom panel), 70% of mice injected with MC38 cells and treated with mNivo exhibited a complete response (Fig. 2C, top panel). Together, the data established that LLC1 is a ‘cold’ tumour model, with a high degree of TAMCs infiltrating the tumour. It did not respond to anti-PD-1 monotherapy but had an abundance of CD200R-expressing myeloid cells. In contrast, MC38 tumours are very immunogenic and responded to mNivo treatment. Nevertheless, some of the mice in this model failed to respond to the therapy and developed tumours. CD200 was expressed on MC38 cells *in vitro* and *in vivo*, suggesting that this could be an additional source of TIIC suppression.

### CD200R and PD-1 blockade in the LLC1 model does not improve efficacy

**Figure 3.**
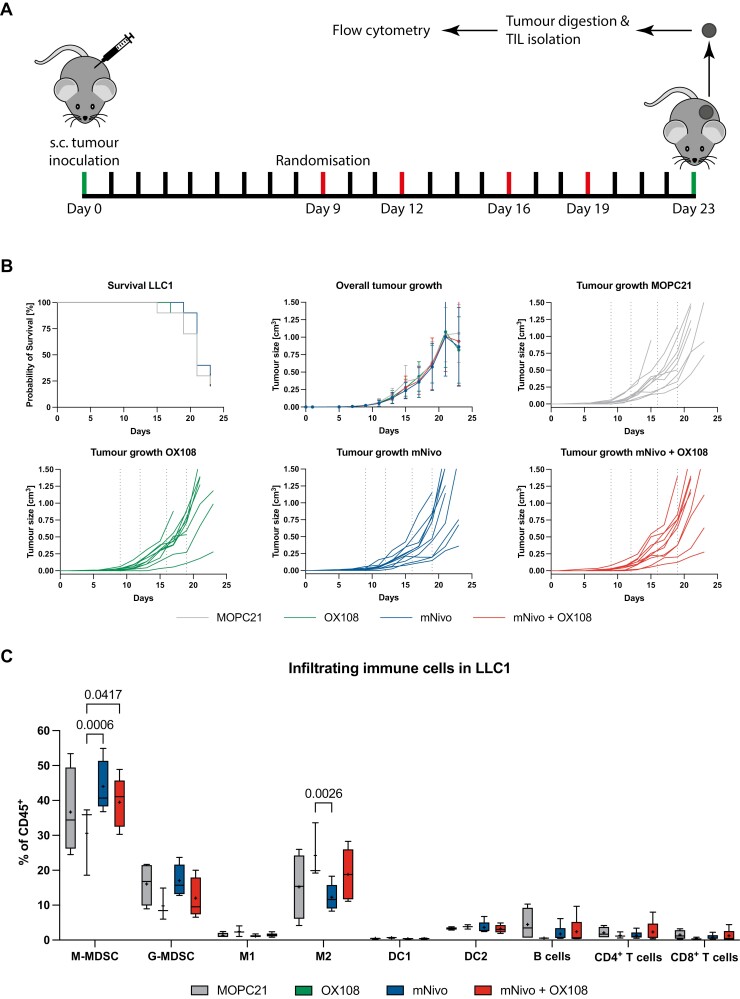
Combination therapy in LLC1 tumours. (A) Illustration describing the workflow for (B) and (C); red lines indicate antibody treatment. (B) LLC1 cells were subcutaneously injected into mice and tumour volume was measured every other day. Once the average tumour volume reached 20 mm^3^, mice were randomised into four different groups and treatment was started. Mice were treated with 200 µg/antibody/treatment, receiving four treatments over 2 weeks. *n* = 10/group. (C) On day 23, all remaining mice were culled, tumours were harvested and digested. Immune cells were enriched using density gradient centrifugation and the abundance of different immune cell populations was analysed. The line shows the median, the ‘+’ shows the mean. Whiskers display the 10–90 percentile.

Having established the response to anti-PD-1 monotherapy, we next sought to test the combination of PD-1 and CD200R blockade. Figure 3A describes the setup of the experiment. Nine days after s.c. injection of LLC1 cells, mice were randomised and allocated to different treatment groups. After the final treatment, tumour growth was monitored for another 4 days; at day 23 the survival experiment was stopped, and tumours were harvested to compare TIICs between the different groups. Combining PD-1 with CD200R blockade did not delay tumour growth, nor did it affect overall survival (Fig. 3B). Nevertheless, to test for potential changes in TIIC composition, the remaining tumours were harvested and analysed. Overall, no clear differences were observed across the different treatment groups (Fig. 3C). While the OX108 monotherapy led to a significant decrease in the abundance of M-MDSC and an increase in M2 macrophages, compared to mNivo monotherapy, this trend was not observed in the combination therapy. This is perhaps explained by the low statistical power due to the small group size of the OX108 treated arm (*n* = 3). No significant correlation was observed after examining individual tumours and comparing TIIC frequencies with tumour volume (data not shown). Furthermore, the ratios of M1/M2 macrophages and CD4^+^/CD8^+^ T cells did not change (Supplementary Fig. S2A). Of note, successful exposure of the tumour to the antibodies was confirmed by a significantly reduced MFI for CD200R when staining with a commercial OX108-PE antibody (Supplementary Fig. S2B). Overall, for the PD-1 therapy-resistant tumour model LLC1, mono- or combination therapy with a CD200R blocking antibody did not improve the overall outcome.

### Lack of improved survival upon CD200R and PD-1 combination therapy in MC38 tumours

**Figure 4.**
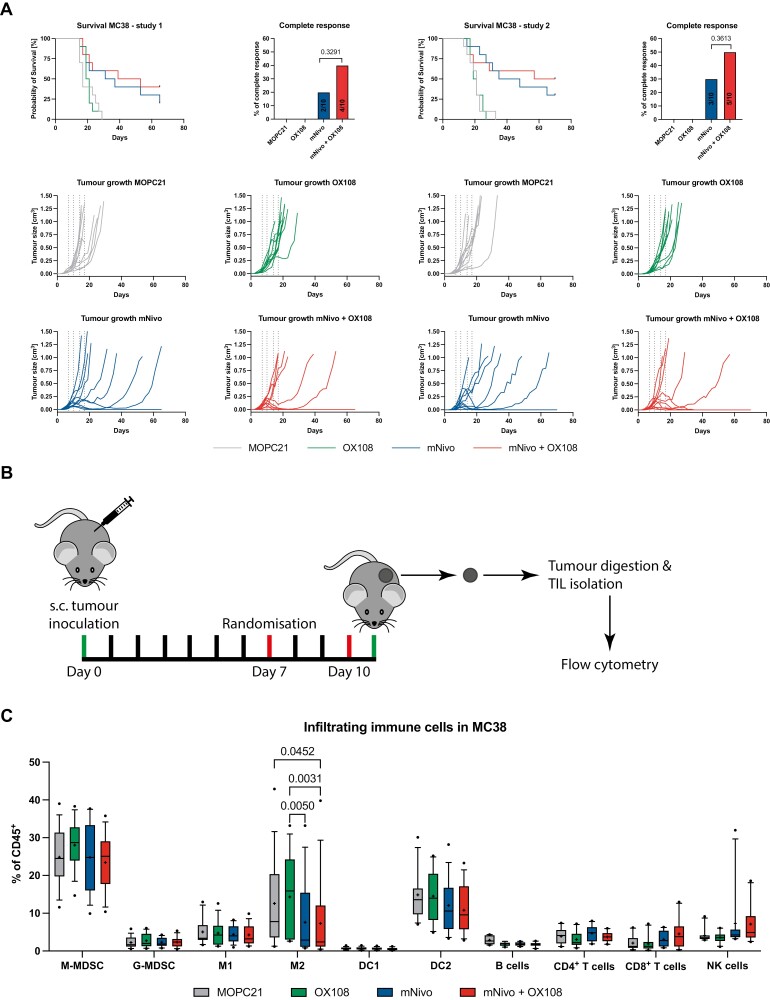
Combination therapy in MC38 tumours. (A) MC38 cells were subcutaneously injected into mice and tumour volume was measured every other day. Once the average tumour volume reached 40 mm^3^, mice were randomised into four different groups and treatment was started. Mice were treated with 200 µg/antibody/treatment, receiving four treatments over 2 weeks. *n* = 10/group. (B) Illustration describing the workflow for (C); red lines indicate antibody treatment. (C) One day after the final treatment, mice were culled, tumours were harvested and digested. Immune cells were enriched using density gradient centrifugation and the abundance of different immune cell populations was analysed. Three independent experiments with *n* = 5/group were pooled. The line shows the median, the ‘+’ shows the mean. Whiskers display the 10–90 percentile.

Lastly, we tested the combination therapy in the PD-1 blockade-sensitive model MC38. Because mice treated with mNivo often had a complete response, the survival study and TIIC analysis were run independently. In the two replicates of the survival study, OX108 monotherapy did not have any effect on tumour growth, and neither was there a significant improvement comparing mNivo monotherapy and the mNivo/OX108 combination therapy (Fig. 4A). In both experiments, however, there was a slight trend towards more complete responses and therefore increased overall survival of mice treated with both antibodies. In the first study, 20% of mice treated with mNivo had a complete response versus 40% treated with both antibodies (*P* = 0.3291). Similarly, in the repeat study, 30% responded to the monotherapy versus 50% responding to the combination therapy (*P* = 0.3613). The trend was reinforced when both studies were combined (*P* = 0.1848), resulting in 5/20 tumour-free mice for anti-PD-1 monotherapy and 9/20 complete responders for the combination therapy (Supplementary Fig. S3A). Importantly, similar results were achieved by a contract research organisation using our mice and antibodies (Supplementary Fig. S4). In this experiment, an additional group treated with mNivo and anti-mCTLA4 (clone 9D9, mIgG2b, BioXCell) was included. Both combination therapies resulted in three tumour-free mice, compared to none in the mNivo monotherapy. As a mouse needed to be excluded from the mNivo/OX108 group due to convulsions, this led to a statistically significant difference in complete responses, compared to mNivo monotherapy (*P* = 0.0466).

To explore a mechanism for these potential effects, we designed an experiment to analyse the TIICs in MC38-treated tumours. As most of the responding tumours started to shrink in the mNivo-treated mice at around day 11, we only treated the mice twice and harvested the tumours the day after the second treatment (Fig. 4B). Each treatment cohort included five mice and this experiment was repeated three times, with all samples being pooled (Fig. 4C). Given that some tumours were expected to completely regress, there was surprisingly little variation in TIICs across the different immune cell subsets. M2 macrophages were significantly increased in OX108-treated tumours, compared to the mNivo- and mNivo/OX108-treated tumours, whereas only the combination therapy led to a significant decrease versus the MOPC21 isotype control. While there was a slight change in the ratio of M1/M2 macrophages in favour of M1 macrophages, the differences were not significant (Supplementary Fig. S2C). Once again, there was a clear reduction in the CD200R-staining MFI in all anti-CD200R antibody-treated tumours (Supplementary Fig. S2D), indicating that the antibody was successfully binding to immune cells in the tumour.

As the data suggested that the combination approach might lead to more complete responses, in one experiment a different flow cytometry panel was used to further distinguish T-cell subsets (naïve, effector, and memory). However, no significant differences were observed (Supplementary Fig. S3B). Furthermore, in two of the three repeats, draining and non-draining lymph nodes (dLN and non-dLN, respectively) were harvested and analysed, but only small differences were observed (Supplementary Fig. S3C and D). There was a significant increase in DC1s in draining, but not non-draining, lymph nodes in the combination therapy. Furthermore, there was a trend towards increased B cells in all treated dLNs, however, this effect was also observed in non-dLNs. Taken together, combining PD-1 and CD200R blocking antibodies did not result in a statistically significant survival benefit in the MC38 tumour model. However, there was a trend towards more complete responses for the mNivo/OX108 antibody combination. Whilst some immune cell populations in the tumour and LN changed, it remains to be determined whether these observations are linked.

## Discussion

In this study, we report the successful use of double knock-in humanised mice to test a new PD-1/CD200R-based combination immunotherapy, using blocking anti-human antibodies of the mouse IgG1 isotype. We confirmed that mNivo (nivolumab mIgG1) retains its affinity for PD-1 and the potential to activate T cells *in vitro*. Upon establishing PD-1 blockade-resistant (LLC1) and -sensitive (MC38) tumour models, combination therapy with mNivo and OX108 was tested. No significant improvement in overall survival was observed for either model, but there was a trend towards more complete responses following combination therapy as well as changes in the immune cell populations infiltrating the MC38 tumours.

It is important to note the limitations of our study. First, CD200 was not expressed on LLC1 cells, but was present on approximately two-thirds of MC38 cells. This might have implied that the CD200/CD200R axis is not a strong contributor to immunosuppression in these models. However, MC38 cells expressed CD200 at levels comparable to PD-L1 and PD-L2 on DCs [35]. Coupled with our observation of CD200R^+^ immune cells within the TME, this suggests that the key elements of the suppressive pathway are present. Additionally, CD200 is also known to be expressed on B and T cells as well as endothelial cells [36], all of which could engage CD200R expressed by TIICs. It is important to note that for the PD-1/PD-L1 axis, the infiltration of CD8^+^ or PD-1^+^ cells has a stronger association with response than PD-L1 expression by tumours per se [37]. Nevertheless, because the colorectal cancer model MC38 expressed PD-L1 as well as CD200, and more complete responses were observed when both receptors were blocked, this supports the possibility that CD200 expression on tumour cells might indeed contribute to the inhibition of TIICs *in vivo*. Therefore, future investigations might profitably focus on tumour models with higher expression of CD200.

An additional caveat is that, in this study, we used two different tumours that differ in the cellular composition of their TMEs, however, both were subcutaneous tumour models. It is likely that orthotopically transplanted tumours, or genetically induced tumours, would develop an entirely different TME. For example, it has been shown that orthotopic and subcutaneous tumours differ in their degree of vascularisation [38], potentially affecting the availability of drugs and the level of immune cell infiltration. Another animal model that could be used is one involving, e.g. a patient-derived xenograft in which the immune system is reconstituted from human stem cells. However, such models are not without their drawbacks as they do not always recapitulate the lymphoid and myeloid compartments equally well [39].

Finally, although OX108 has a relatively low affinity (K_D_ = 84 nM), it is a well-documented CD200R-blocking antibody [24], and we were able to confirm successful tumour penetration and binding via a reduction of CD200R MFI on TIICs. Nevertheless, higher-affinity monoclonal antibodies targeting CD200R should also be tested, as affinity is claimed to correlate with efficacy [40].

Overall, we found several significant changes in immune cell infiltration. However, in the LLC1 tumours, the observed differences in TIICs need to be treated with caution owing to the large variation in the data. In contrast, the reduction of M2 macrophages in MC38 tumours upon treatment with mNivo or the mNivo/OX108 combination was observed for all three biological repeats with *n* = 5/group. Whereas the combination therapy reduced M2 macrophages significantly compared to MOPC21 (*P* = 0.0452), the mNivo monotherapy failed to do so, indicating a potential benefit of OX108 co-treatment. In contrast, anti-CD200R monotherapy did not change the M2 macrophage abundance compared to the isotype control (*P* = 0.8232). Importantly, anti-PD-1 monotherapy produced a clear trend towards reduced M2 macrophages overall, while failing to reach significance when compared to MOPC21 (*P* = 0.0654). As mNivo and mNivo/OX108 treatment were very similar (*P* = 0.9990), it is more likely that the observed effects are driven mostly by mNivo, rather than OX108. It was surprising, however, that there was no significant increase in the number of CD8^+^ T cells, as this was previously observed for the MC38 model treated with PD-1 blocking antibodies [29, 30]. These discrepancies are potentially explained by the different time points chosen for the TIIC analysis in each study. In the present study, tumours were harvested significantly earlier (day 11) compared to the published studies (day 15 and day 16). Therefore, increased CD8^+^ T cell infiltration might only be observed at a later stage. Whilst the survival analysis did not reveal significant differences, there was a trend towards more complete responses following anti-PD-1/CD200R combination therapy, compared to anti-PD-1 alone. This prompted an investigation of the T-cell subsets in the tumour and dLN (data not shown) as well as a general characterisation of the immune cells within the dLN and non-dLN. The increase in DC1s in the dLN is of particular interest as DC1s are required for antigen trafficking and priming of CD8^+^ T cells [41]. However, it should also be noted that the error associated with measuring these rare immune cell subsets was significant, meaning that follow-up studies would be needed to confirm these observations.

The precise role of CD200R-immunoregulation in cancer remains elusive and there are contradictory results concerning whether, overall, CD200R is pro-tumourigenic or tumouricidal. While this study tested the potential of the antagonistic clone OX108 in combination with mNivo, other mouse studies suggest that inducing signalling by CD200R enhances tumour clearance. Whereas CD200 knockdown by shRNA significantly reduced lung metastasis [42] and CD200^-/-^ mice exhibited increased resistance to chemically induced papillomas [17], reduced metastasis burden in the 4THM breast cancer model in mice overexpressing CD200 has also been reported [18]. Similarly, Talebian *et al.* [13]. found that B16 melanoma cells expressing OVA and CD200 exhibited diminished primary tumour growth as well as metastasis compared to B16-OVA cells. Subsequently, in a follow-up study using Yumm1.7 melanoma cells, Talebian *et al*. demonstrated reduced immune cell infiltration and increased tumour growth in mice lacking CD200R [43]. Interestingly, no effect on tumour growth was observed following treatment with anti-CD200 antibodies. Those reports contrast with a recent study comparing EMT6, LLC1, and B16 cells [15], wherein agonistic anti-CD200R antibodies had no effect on tumour growth, and B16 tumours grew more slowly in CD200^-/-^ mice than in WT mice.

In contrast to the conflicting mouse studies, most reports investigating the role of CD200R in humans support a pro-tumourigenic role of CD200R. CD200 was shown to be upregulated in a great variety of haematological malignancies [44] and solid tumours [45]. Elevated CD200 expression has also been linked to decreased numbers of CD8^+^ T cells and increased regulatory T cells [46]. For NOD/SCID mice injected with human lymphoma cells expressing CD200 and treated with an anti-CD200 antibody without effector functions, i.e. a ‘pure’ blocking antibody, there was superior tumour growth inhibition versus an antibody with effector functions [47]. This led to the development of samalizumab (ALXN6000), a first-in-class anti-CD200 antibody tested in phase I clinical trials for multiple myeloma and chronic lymphocytic leukaemia by Alexion Pharmaceuticals [48]. In 2022, 23andMe announced the development of an anti-CD200R blocking antibody for use in solid cancers based on the findings of their proprietary genetic and health survey database [49], and a phase I clinical trial is now underway [50].

Here we tested the potential of combining anti-CD200R and anti-PD-1 immune checkpoint blockade, since (i) the human studies clearly suggested a tumour-promoting role of CD200R, (ii) most mouse studies describing tumouricidal effects of CD200R relied on examining the effects of gene deletion or over-expression rather than antibody-based interventions, and (iii) a majority of reports focused on either single gene perturbations or antibody monotherapies. To remove the potential risk of depleting CD200R^+^ or PD-1^+^ immune cells, the D265A mutation [25] was used to reduce the Fc effector function and create ‘pure’ blocking antibodies. Significant effects on survival in the LLC1 and MC38 models were not observed, but a tendency towards more complete responses in the MC38 model warrants further investigation.

## Supplementary Material

ltad006_suppl_Supplementary_Material

## Data Availability

The data underlying this article are available in the article and in its online supplementary material. All materials described in this manuscript are available upon request.

## Abbreviations

CD200R: CD200 receptor
DC: dendritic cell
dLN: draining lymph node
FcγR: Fc gamma receptor
G-MDSC: granulocytic myeloid-derived suppressor cell
MFI: median fluorescence intensity
M-MDSC: monocytic myeloid-derived suppressor cell
mNivo: nivolumab mIgG1
PD-1: programmed cell death protein 1
scFv: single-chain variable fragment
SPR: surface plasmon resonance
TAMC: tumour-associated myeloid cell
TCS: T-cell stimulator
TIIC: tumour-infiltrating immune cell
TME: tumour microenvironment

## Permission to reproduce

This work may be reproduced with the permission of the corresponding author.

## Clinical trial registration

N/A.
