# Supplementary material for: Combination CD200R/PD-1 blockade in a humanised mouse model
Source: Immunother Adv. 2023 Mar 30;3(1):ltad006. doi: 10.1093/immadv/ltad006 (PMC10112683; doi:10.1093/immadv/ltad006)
Supplement: ltad006_suppl_Supplementary_Material [file ltad006_suppl_supplementary_material.docx]

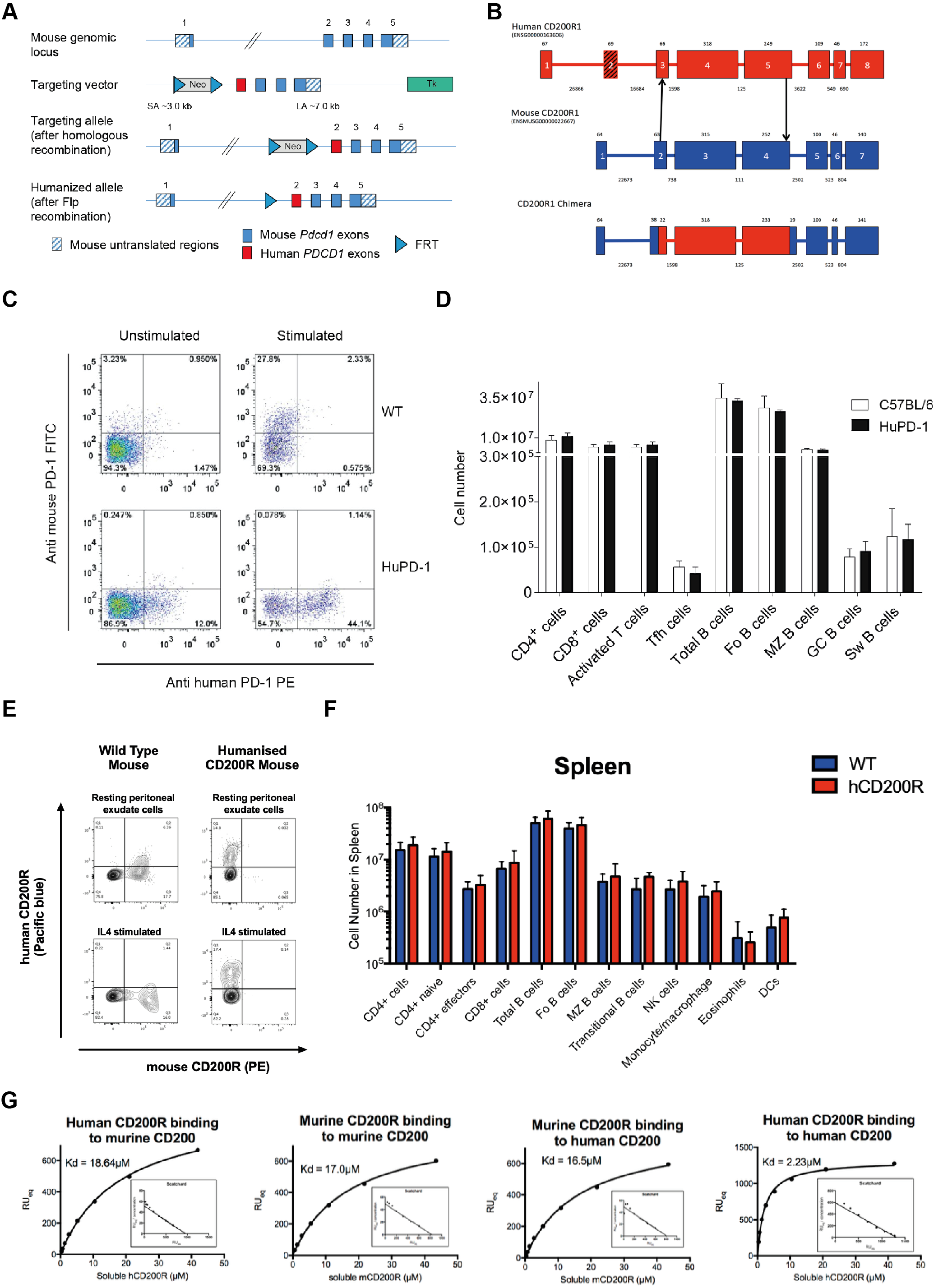


Figure S1 Generation and validation of humanised PD-1 and CD200R mice
(A) Strategy for generation of the humanised allele in humanised PD-1 mice. Murine *Pdcd1* exon 2, encoding the extracellular domain of PD-1, was replaced by the human counterpart using targeted homologous recombination by electroporation of mouse C57BL/6 embryonic stem cells. Microinjection of blastocytes with positive embryonic stem cell clones was performed with implantation of blastocytes into foster mice. Chimeric offspring were bred to FLP transgenic mice and offspring with germline transmission of the knock-in, plus somatic deletion of the FRT flanked neomycin resistance cassette, were identified. Mice were bred to homozygosity of the humanised allele, with genotyping performed by PCR of digested ear punch samples with primers specific for human or mouse exons. (B) As in (A) but for the human *CD200R* exons 3-5. Human exon 2 is alternatively spliced and likely not expressed at a protein level [51]. To maintain appropriate splicing the murine sequence is retained at each end of the inserted exons. (C) Expression of PD-1 on wild type and humanised PD-1 T cells upon *in vitro* stimulation with 1 µg/ml anti-CD3 antibody. (D) Comparison of the naïve status of humanised PD-1 and C57BL/6 immune systems. Quantification of immune cell subsets in spleen by flow cytometry, gating for specific populations was as follows: activated T cells (CD3^+^CD44^+^ICOS^+^), Tfh (CD4^+^ICOS^+^CXCR5^+^), Fo B cells (B220^+^CD23^hi^CD21^int^), MZ B cells (B220^+^CD23^lo^CD21^hi^), GC B cells (B220^+^CD95^+^GL7^+^), Sw B cells (B220^+^IgG^+^). Data were pooled from three independent experiments with at least two mice per group (mean ± SD). (E) Expression of CD200R on wild type and humanised CD200R mouse peritoneal exudate cells at rest and following activation with IL-4. (F) Comparison of the naïve status of humanised CD200R and C57BL/6 immune systems. Quantification of immune cell subsets in spleen by flow cytometry, gating for specific populations was as follows: CD4^+^ T cells (CD4^+^), CD4^+^ naïve (CD4^+^CD62L^+^CD44^-^), CD4^+^ effectors (CD4^+^CD44^+^CD62L^-^), CD8^+^ cells (CD8^+^), total B cells (B220^+^CD19^+^), Fo B cells (B220^+^CD19^+^CD21^+^CD23^+^), MZ B cells (B220^+^CD19^+^CD21^+^CD23^-^), transitional B cells (B220^+^CD19^+^CD93^+^), NK cells (NK1.1^+^CD3^-^), monocyte/macrophage (B220^-^CD3^-^CD11b^+^CD11c^-^Ly-6G^-^SSC^low^), eosinophils (B220^-^CD3^-^CD11b^+^CD11c^-^Ly-6G^-^SSC^high^), DCs (B220^-^CD3^-^CD11b^+^CD11c^+^Ly-6G^-^). (G) Cross-species CD200R-CD200 interaction was assessed by SPR at 37 °C. Soluble monomeric human or mouse CD200R was injected over immobilised human or mouse CD200 at various concentrations. Double-referenced binding at equilibrium was plotted against analyte concentration and the K_D_ for each interaction calculated by curve fitting. The inset Scatchard plots are a linear transformation of the data that allow visual inspection of the quality of fit to the calculated K_D_.

**References**
51. Vieites JM, Torre R de la, Ortega MA *et al.* Characterization of human cd200 glycoprotein receptor gene located on chromosome 3q12-13. *Gene* 2003;311:99–104., DOI: 10.1016/s0378-1119(03)00562-6


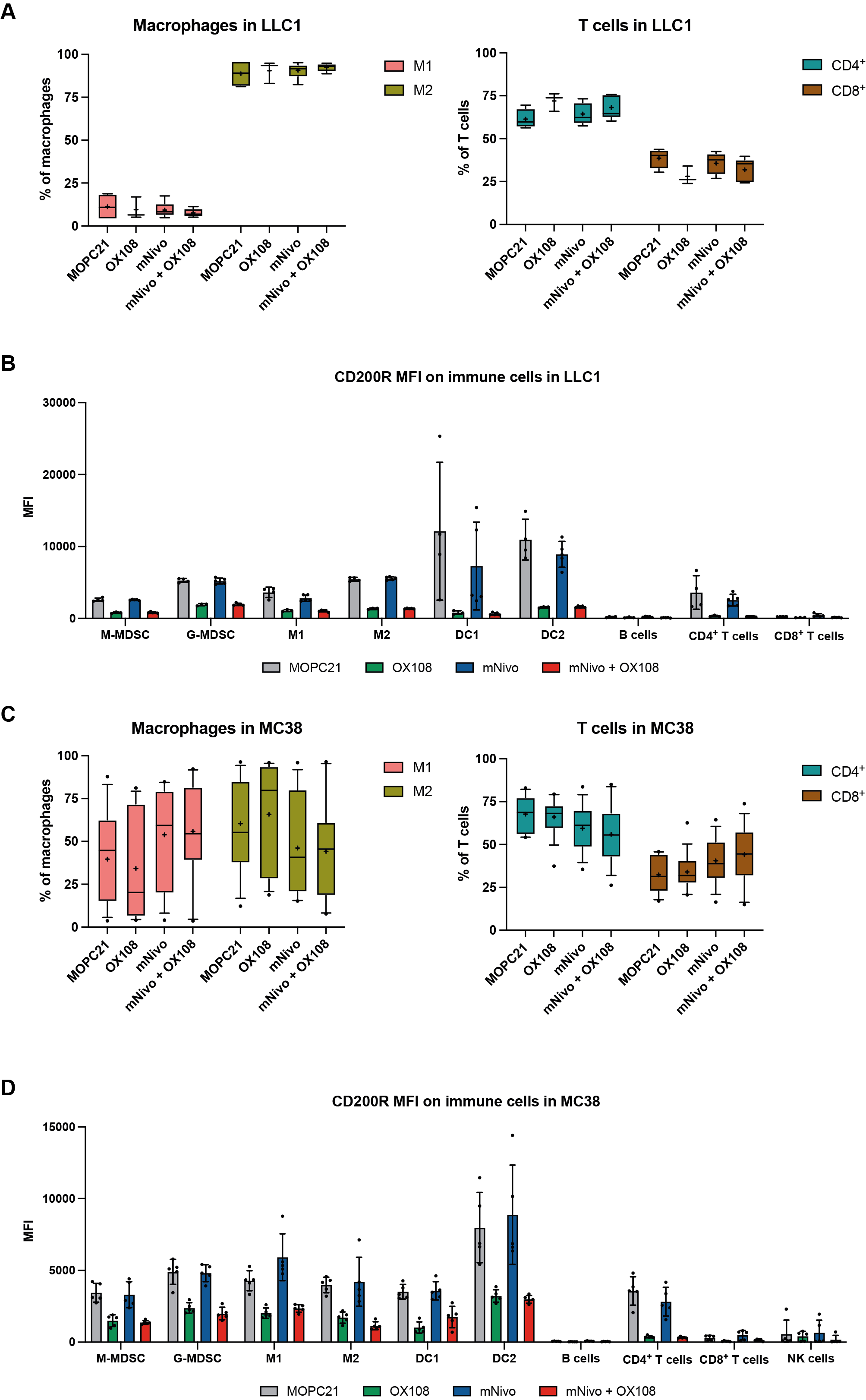


Figure S2 Combination therapy in LLC1 and MC38 tumours
(A + B) Protocol as described in Figure 3C. Ratios of M1/M2 macrophages (A, left panel) and CD4^+^/CD8^+^ T cells (A, right panel) as well as MFI of CD200R (B) are shown. (C + D) Protocol as described in Figure 4C. Ratios of M1/M2 macrophages (C, left panel) and CD4^+^/CD8^+^ T cells (C, right panel) as well as MFI of CD200R (D) are shown. For boxplots, the line shows the median, the ‘+’ shows the mean. Whiskers display the 10-90 percentile. For bar graphs, data are shown as mean ± SD.


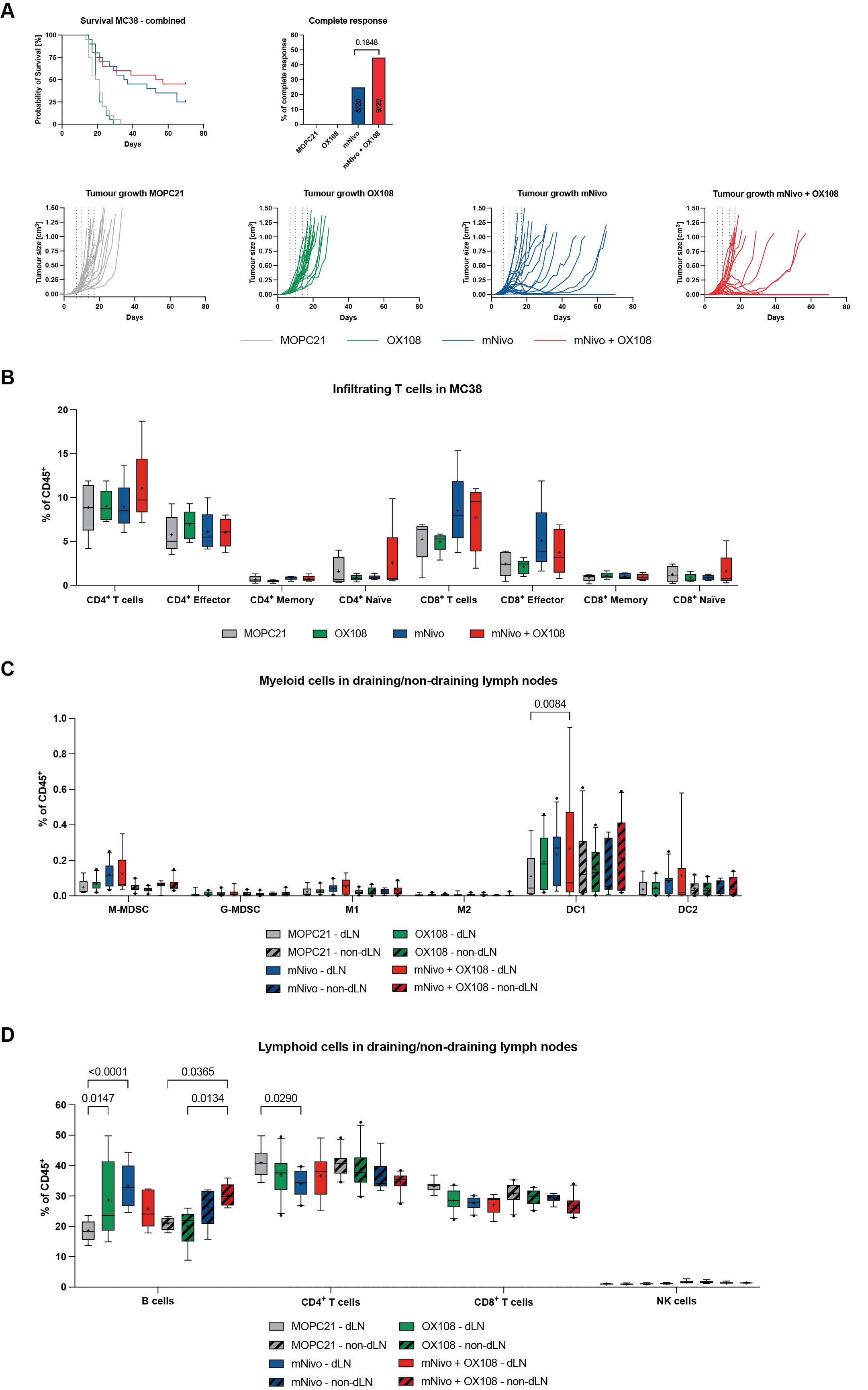


Figure S3 Combination therapy in MC38 tumours
(A) Data from Figure 4A was pooled. (B) Protocol as described in Figure 4C. Different T-cell subsets were analysed by flow cytometry. (C + D) One day after the final treatment, mice were culled, draining and non-draining lymph nodes were harvested and digested. Abundances of myeloid (C) and lymphoid (D) immune cell populations were analysed. The line shows the median, the ‘+’ shows the mean. Whiskers display the 10-90 percentile.


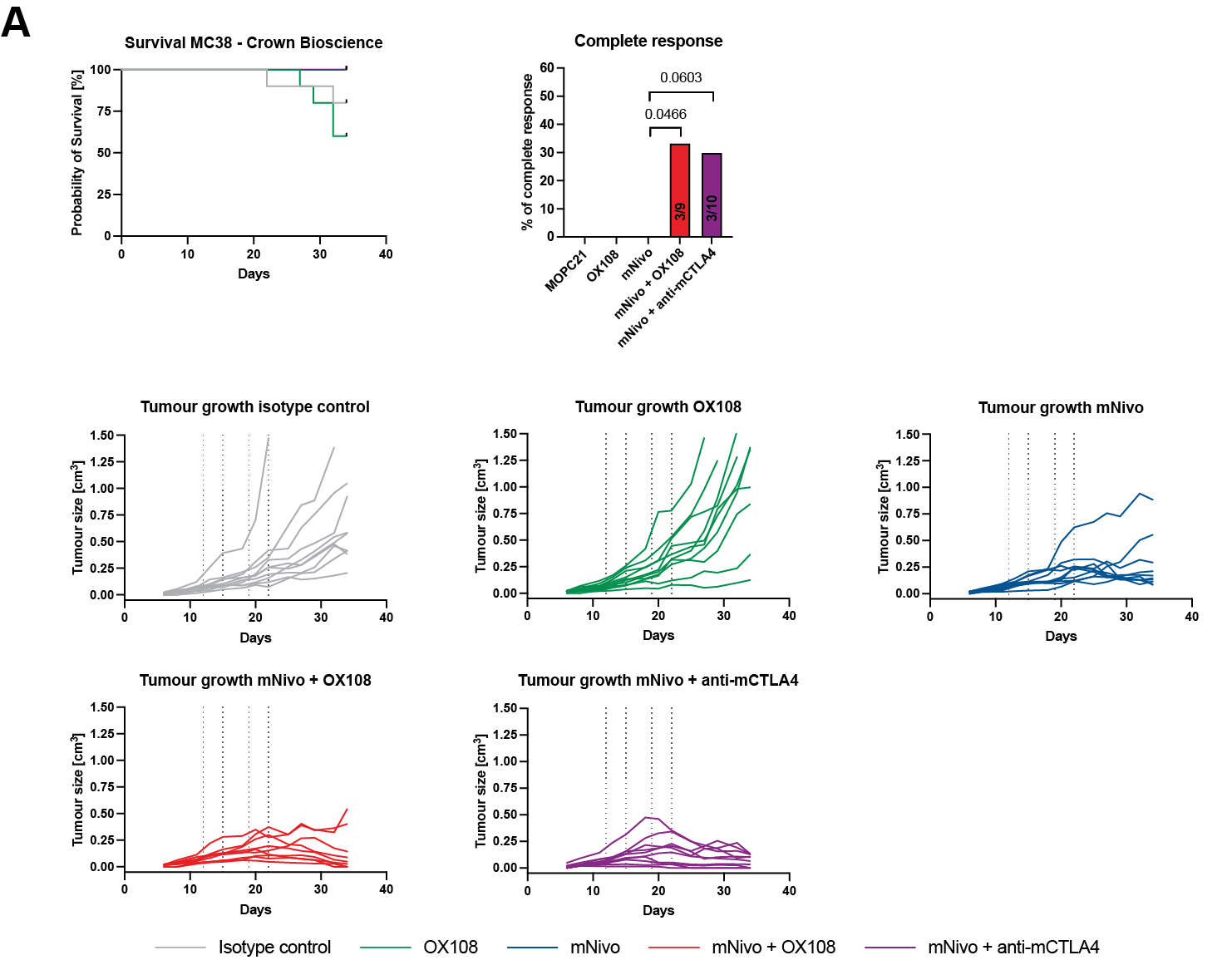


Figure S4 Combination therapy in MC38 tumours by Crown Bioscience
(A) 1 × 10^6^ MC38 cells were subcutaneously injected into mice and tumour volume was measured three times a week. On day 11 (average tumour volume 58 mm^3^), mice were randomised into groups and treatment was started the subsequent day. Mice were treated with 10 mg/kg per antibody/treatment, receiving four treatments over two weeks. n = 10/group; one mouse in the mNivo/OX108 group was excluded from the study at day 12 due to convulsions.
This experiment was carried out by the contract research organisation Crown Bioscience using our mice and antibodies.


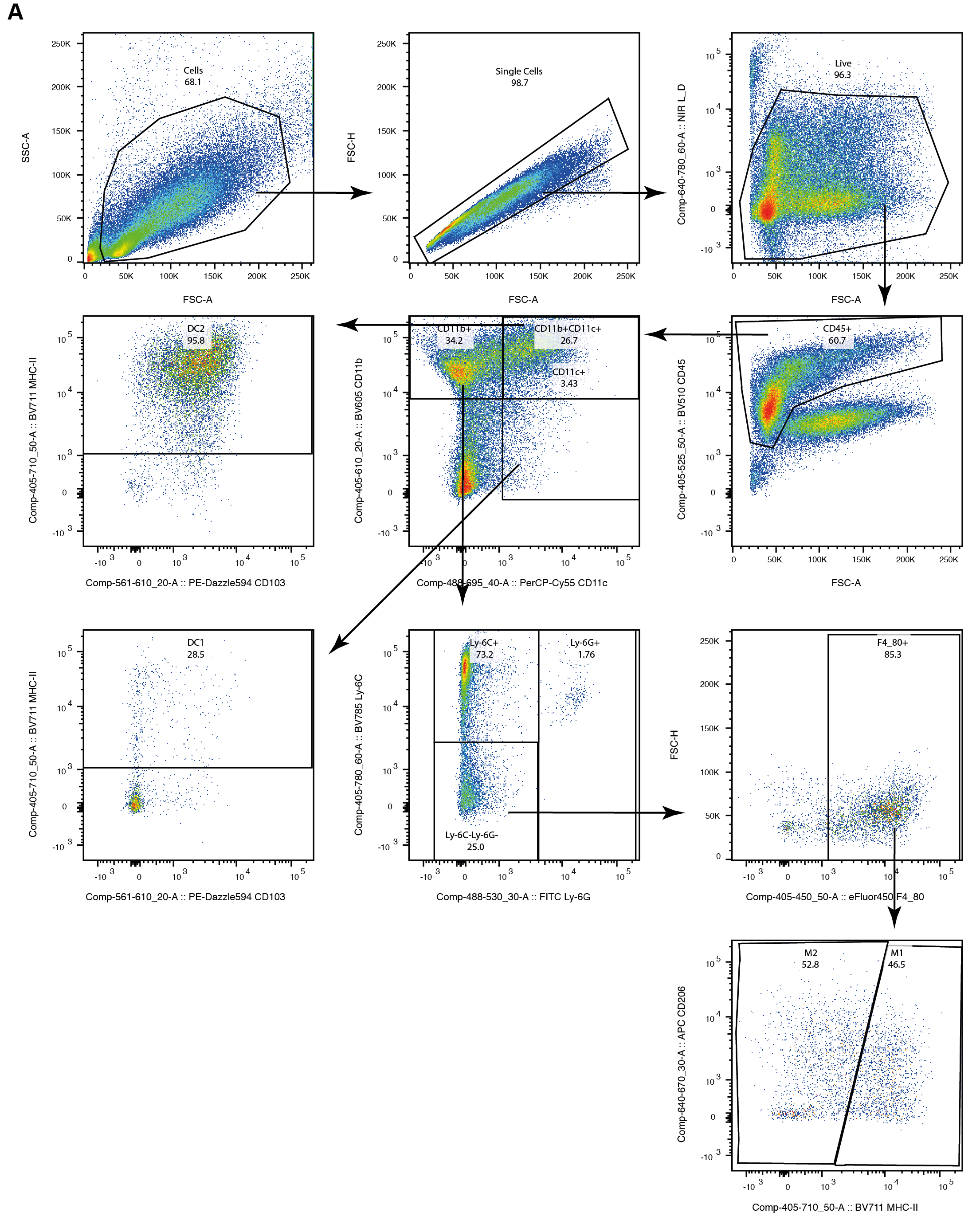


Figure S5 Gating strategy for myeloid immune cells
(A) Different myeloid immune cell subsets were identified using following markers: Live/Dead, CD45, CD11b, CD11c, Ly-6C, Ly-6G, F4/80, CD206, MHC-II.


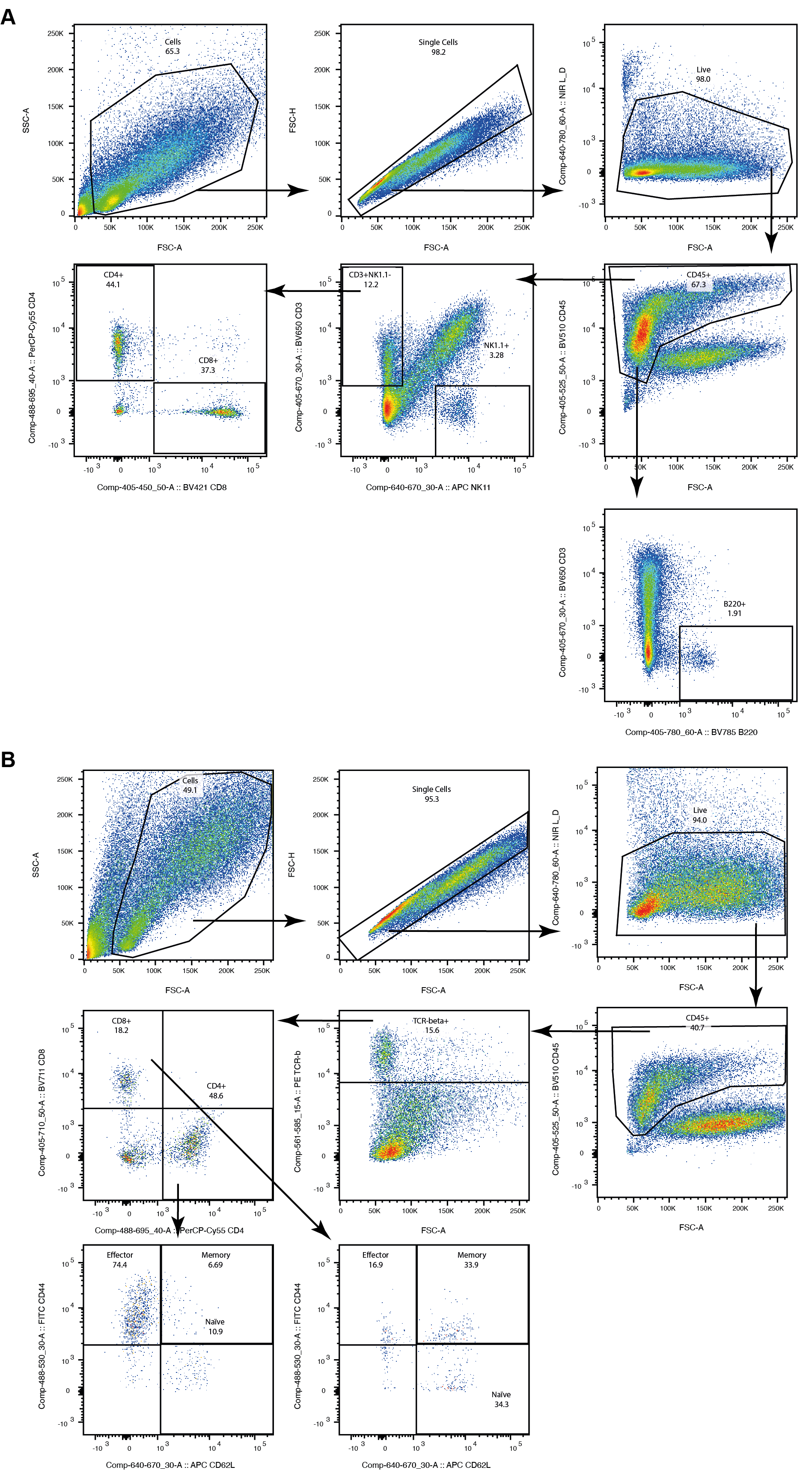


Figure S6 Gating strategy for lymphoid immune cells and T-cell subsets
(A) Different lymphoid immune cell subsets were identified using following markers: Live/Dead, CD45, CD3, NK1.1, B220, CD4, CD8. (B) Different T-cell subsets were identified using following markers: Live/Dead, CD45, TCR-β, CD4, CD8, CD44, CD62L.
